# Localization-based techniques for super-resolution imaging of vascular dynamics

**DOI:** 10.1016/j.xinn.2026.101401

**Published:** 2026-04-15

**Authors:** Quanyu Zhou, Daniil Nozdriukhin, Zhenyue Chen, Xosé Luís Deán-Ben, Daniel Razansky

**Affiliations:** 1 Institute for Biomedical Engineering and Institute of Pharmacology and Toxicology, Faculty of Medicine, University of Zurich, 8057 Zurich, Switzerland; 2 Institute for Biomedical Engineering, Department of Information Technology and Electrical Engineering, ETH Zurich, 8093 Zurich, Switzerland; 3 Institute of Precision Optical Engineering, School of Physics Science and Engineering, Tongji University, Shanghai 200092, China

The mammalian vascular system is a marvel of biological engineering, comprising an extensive network of vessels that sustains every organ and tissue in the body. Within this network, microvessels smaller than 100 μm—including capillaries, arterioles, and venules—are the principal sites of exchange for oxygen, nutrients, and waste. Yet, their minute scale and structural complexity have long impeded detailed observation, leaving the role of microcirculation unresolved in key pathological processes underlying diseases such as cancer, neurovascular disorders, diabetic microangiopathy, and chronic inflammation (Figure 1A).

**Figure 1:**
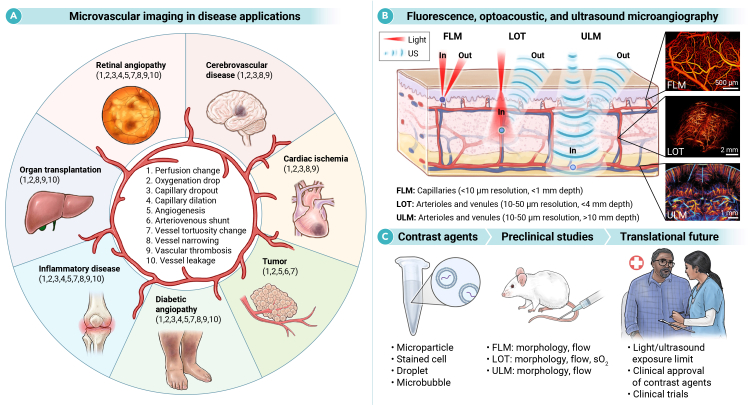
Imaging principles and applications of localization-based microangiography (A) Examples of vascular alterations (numbered 1–10) accessible to localization microangiography across diverse vascular disorders. (B) Principles of FLM, LOT, and ULM, along with their achievable spatial resolution and penetration depth. Representative images were adapted from Zhou et al..,^1^ Couture et al.,^2^ and Nozdriukhin et al.^3^ Reprinted with permission.^2^ Copyright 2018, IEEE. (C) Overview of contrast agents, preclinical readouts, and translational routes.

For decades, our ability to visualize microvascular structures and functions has remained a formidable challenge. Conventional angiographic techniques, including X-ray angiography, Doppler ultrasound, magnetic resonance imaging, fluorescence microscopy, optical coherence angiography, optoacoustic microscopy and tomography, and intravascular methods, provide access to vascular anatomy and dynamics, but their capabilities remain constrained by tissue motion and resolution-depth trade-offs.

Recently, a new toolset of localization-based microangiography methods leveraging fluorescence, ultrasound, and hybrid optoacoustic contrast has emerged, breaking long-standing resolution-depth barriers and offering unprecedented noninvasive quantification of vascular anatomy and blood flow down to the capillary scale. These approaches are inspired by single-molecule localization microscopy (SMLM), a Nobel Prize-awarded method that surpasses the optical diffraction limit (∼200–300 nm). In contrast to SMLM, which relies on stochastically photoactivated yet static fluorescent molecules in superficial or transparent samples, localization-based microangiography exploits flowing exogenous particles or cells in vessels as sparse emitters. Precise localization overcomes intrinsic diffraction- or diffusion-related resolution limits in deep, living tissues while enabling simultaneous extraction of blood flow velocity and direction via particle tracking. By harnessing different contrast mechanisms (fluorescence, acoustic scattering, and optical absorption), these methods feature complementary advantages in resolution, depth, and application domains (Figure 1B). Here, we outline how these emerging methods uncover fundamental mechanisms underlying microvascular dysfunction and lay the groundwork for next-generation diagnostic tools.

## Wide-field fluorescence localization methods

Leveraging an expanding toolbox of fluorophores, wide-field fluorescence localization microscopy (FLM) can interrogate the microvascular network via intravenous injection of fluorescent particles or labeled cells.^1^ These circulating probes act as sparse point emitters that can be imaged using wide-field systems equipped with high-speed cameras, enabling localization and tracking with ∼5 μm precision, a manyfold improvement compared to the resolving capacity imposed by intense light diffusion in deep tissues. Hence, vascular architecture and hemodynamic readouts (e.g., flow velocity and direction) can be mapped in exquisite detail to resolve the smallest capillary networks (<10 μm) in the horizontal/transverse plane, particularly advantageous for visualizing cerebrovascular networks in cortical brain areas. Although wide-field imaging intrinsically lacks depth information, 3D localization can be achieved through point spread function engineering or multi-view imaging. The performance of FLM is fundamentally constrained by light propagation in biological tissues. Experimentally validated imaging depths typically lie in the 200–500 μm range in the visible and first near-infrared (NIR-I; 650–1,000 nm) spectral windows, increasing to 0.6–1 mm in the second NIR (NIR-II; 1,000–1,700 nm) window owing to reduced scattering.

## Ultrasound localization microscopy

Ultrasound localization microscopy (ULM) exploits the low tissue attenuation of ultrasound for deep microvascular imaging. By localizing intravenously injected lipid- or polymer-shelled microbubbles,^2^^,^^4^ ULM surpasses the acoustic diffraction limit by approximately one order of magnitude and is commonly implemented with linear array transducers, thus providing sagittal/coronal cross-sectional views for brain imaging. Alternatively, volumetric imaging can be achieved with matrix/row-column transducers. Center frequencies in the 2–15 MHz range are typically selected depending on the imaging target. Ultrafast plane-wave transmission at kilohertz rates enables the tracking of fast-flowing microbubbles, while multi-angle compounding improves the signal-to-noise ratio (SNR). Unlike FLM, ULM requires pre-filtering of raw image sequences using algorithms such as singular value decomposition to suppress static tissue background and enhance acoustic backscatter from individual microbubbles. Subsequent localization and tracking render high-resolution mapping of vascular structure and blood flow dynamics. ULM is distinguished by the use of FDA-approved microbubble contrast agents, compatibility with commercial ultrasound systems, and deep-tissue penetration. Ongoing advances in high-throughput acquisition electronics and motion compensation algorithms continue to expand its capabilities and translational potential.

## Localization optoacoustic tomography

Optoacoustic imaging overcomes light diffusion limits by combining pulsed laser excitation with ultrasound detection, enabling deep-tissue visualization. Building on this, localization optoacoustic tomography (LOT) surpasses acoustic diffraction limits by tracking optically absorbing particles, such as dye-loaded microdroplets or metallic nanoparticles, that can be distinguished from the strong blood background.^3^ Unlike ULM, LOT commonly employs hemispherical ultrasound arrays with extensive angular coverage for rapid 3D particle isolation and tracking. The center frequency depends on the size of the imaged object and usually lies in the 4–10 MHz range when imaging the rodent brain. By combining multi-wavelength excitation with spectral unmixing, optoacoustics uniquely enables simultaneous mapping of vascular architecture, flow dynamics, and tissue oxygenation, beyond what pure optical or acoustic modalities can achieve. LOT has demonstrated ∼4 mm penetration with down to 10 μm resolution, well beyond the diffraction-limited resolution of ∼150 μm for 5 MHz detection bandwidth. However, real-time 3D imaging is constrained by trade-offs between the laser pulse repetition rate and per-pulse energy, while deeper penetration is constrained by reduced SNR due to light fluence decay. Ongoing efforts focus on improving light delivery through advanced guiding systems and optimizing contrast agents with enhanced absorption cross-sections.

## Preclinical and translational applications

In preclinical research, FLM enables transcranial imaging of cortical vasculature in mice, resolving vessels from large pial branches to fine capillaries and providing multiparametric readouts during ischemic stroke. LOT features superior penetration depth and 3D imaging capability, enabling visualization of pial and penetrating microvascular alterations through the scalp and skull in mouse models of neurovascular diseases and brain tumors. ULM has been more broadly applied in animal models to map microvascular networks and flow dynamics across organs and diseases, including ischemic stroke, diabetic pancreas and kidneys, coronary microcirculation, and tumors. All localization methods are limited by temporal resolution, as vascular reconstruction requires sufficient emitter accumulation over time amid rapid clearance of particles/microbubbles from circulation. Continuous microbubble infusion and the use of labeled blood cells have been shown to sustain stably circulating emitters, enabling a 1 Hz effective frame rate and quantification of functional hyperemia during sensory stimulation. Beyond preclinical studies, ULM has proven transformative for microvascular assessment in human liver, breast, kidney, heart, and brain, including transcranial cerebrovascular imaging through the temporal bone with microscopic-resolution views.^5^

Despite this progress, several barriers remain for clinical adoption of localization microangiography, including technical limitations, contrast agent biosafety, and clinical validation (Figure 1C). Optical methods are depth limited by photon scattering and absorption, which can be partially mitigated by operating in the NIR-II window or via optical clearing. ULM achieves centimeter-scale penetration but struggles with bone/air interfaces and limited signal strength generated by microbubbles, which can be alleviated through advanced beamforming, compounding, and aberration correction. Contrast agent safety represents another key hurdle. While ultrasound agents have regulatory approval for clinical use, most optical agents are predominantly experimental. Even when employing clinically approved dyes such as indocyanine green (ICG), new particle formulations must be validated in long-term toxicity studies. Clinical validation further necessitates benchmarking against gold-standard techniques. The clinical trial pathway is most advanced for ULM, whereas FLM and LOT translation remains dependent on further technical innovations.

Overall, the field is progressing rapidly, with ULM leveraging existing clinical infrastructure and approved agents and FLM benefiting from low-cost optical components and superior resolving capacity. LOT is seeing cost reductions through laser diode advances, with optoacoustic imaging systems recently achieving clinical certification. This multi-modal landscape suggests that localization-based optical and acoustic imaging techniques will likely find distinct application niches depending on depth, resolution requirements, target anatomy, and functional readouts.

## Funding and acknowledgments

The authors acknowledge grant support from the 10.13039/501100001711Swiss National Science Foundation (310030_192757, 10.006.824, and 10.003.762), the 10.13039/501100003006ETH Zürich grants (ETH-C-01 21-2 and ETH-033 25-1), the 10.13039/501100013348Innosuisse - Swiss Innovation Agency (51767.1 IP-LS), the Personalized Health and Related Technologies (PHRT) of the ETH Domain (PHRT-582), the United States National Institutes of Health (RF1-NS126102), the EU Joint Programme – Neurodegenerative Disease Research (JPND2022-083, *AUTOFUS*), the Swiss Cancer Research, (KFS-5234-02-2021 and KFS-6361-02-2025), and the Swiss State Secretariat for Education, Research, and Innovation (SERI) under Horizon Europe MSCA Doctoral Network grant (101119924, *BE-LIGHT*) and European Horizon RIA Digital and Emerging Technologies grant (101135053, *SWEEPICS*). The authors thank V. E. Fulford from Alar Illustration for her scientific illustrations. The funders had no role in the study design, data collection and analysis, decision to publish, or preparation of the manuscript.

## Declaration of interests

The authors declare no competing interests.
